# Early serum progesterone and prolactin analysis at day 9 of oocyte retrieval as a predictor of success in fresh ICSI cycles

**DOI:** 10.5935/1518-0557.20180008

**Published:** 2018

**Authors:** Paulina A. Santander Pérez, Álvaro P. Ceschin, Daniela M P de Moraes, Lucileine K S N de Oliveira, Ianae I Ceschin, Nathan I Ceschin

**Affiliations:** 1 Feliccità Instituto de Fertilidade, Curitiba, Brazil

**Keywords:** early serum progesterone, prolactin, oocyte retrieval

## Abstract

**Objective:**

To analyze progesterone and prolactin plasma levels nine days after oocyte retrieval and evaluate their correlation with pregnancy rates in *in vitro* fertilization cycles. To achieve pregnancy, several factors are analyzed before and during the *in vitro* fertilization cycle. Progesterone supplementation for adequate luteal phase support is indicated despite the presence of multiple corpus luteum in IVF stimulation cycles because of blockage caused by hypothalamic agonists and antagonists. The dosage of progesterone and prolactin on day 09 after follicular retrieval could function as a predictive marker of success in fertility treatments.

**Methods:**

A retrospective study was performed using data from 238 patients submitted to intracytoplasmic sperm injection (ICSI) at a private infertility clinic from January 2013 to December 2015. Hormonal measurements were performed on day 09 after follicular uptake. The data was compared to assess the correlation between prolactin and progesterone dosages and pregnancy rates.

**Results:**

The ICSI pregnancy rate was 40.8% (n=238). No statistically significant difference was observed when correlating the success of the procedure with the prolactin dosage (*p*=0.71). However, progesterone showed a significant difference (*p*=0.021). The cutoff point, indicated by the ROC curve fit according to which gestation would be identified, is 25.95ng/ml of progesterone. The sensitivity of this point is 61.9% and the specificity is 57.4%.

**Conclusion:**

Progesterone dosage may be one of the indicators of gestation on day 09 after follicular uptake. Such data can help physicians to monitoring and provides suitable early gestational care. More studies are needed to corroborate the data found.

## INTRODUCTION

Several studies have attempted to determine cut-off points with laboratory tests that can predict the success of fertilization cycles before serum analysis of hCG. These analyses can be also used to adjust the doses of progesterone administered during the luteal phase, because the prediction of pregnancy success is important for both couples and professionals working whit *in vitro* fertilization cycles (IVF) (Ioannidis *et al*., 2005).

For successful implantation, healthy embryos, adequate endometrium and a functional corpus luteum are required to maintain this receptive endometrium. In both natural and induced cycles, the corpus luteum plays a preponderant role in the onset of gestation, since its primary function is to secrete progesterone to induce the secretory transformations of the endometrium, favoring embryo fixation. The corpus luteum is formed after ovulation, and kept in operation by constant stimulation of the luteinizing hormone (LH). From the physiological point of view, in the process of luteinization of the theca and granulosa cells, attenuation of follicle stimulating hormone (FSH) receptors occurs, transient decrease of the receptors for LH and prolonged stimulation of the receptors for prolactin (PRL) (Stocco *et al*., 2007). Thus, the maintenance of this corpus luteum depends on normal LH pulses every 4 to 6 hours and adequate concentrations of prolactin, since this seems to increase progesterone synthesis (Medeiros, 2014).

After embryo implantation, this effect continues to occur through the action of human chorionic gonadotropin (hCG) on the LH receptors of the corpus luteum, which then remains in operation to maintain the early stages of pregnancy. Thus, in natural cycles, it is known that progesterone production is exclusive to the corpus luteum until the seventh week of gestation. Between seven and nine weeks, the production is made by both the corpus luteum and the trophoblast. After nine weeks, production is almost entirely derived from the trophoblast (Csapo *et al*., 1973).

Prolactin (PRL) could also interfere with the luteal phase. Corroborating this hypothesis, it was demonstrated that prolactin production would be deficient in the secretory endometrium that presented a luteal phase defect compared to the production of a normal secretory endometrium (Daly *et al*., 1981). Thus, it was suggested that the analysis of prolactin in the middle of the Luteal phase could be useful in determining IVF outcome. However, the role of prolactin in the implantation and maintenance of pregnancy after IVF is still uncertain (Ioannidis *et al.*, 2005).

### Objectives

The objective of this project was to analyze plasma levels of progesterone and prolactin on day 09 after follicular uptake and to evaluate their correlation with the success rates of *in vitro* fresh fertilization cycles performed in the private clinic.

## MATERIAL AND METHODS

### Patients

This analysis included data records of 322 patients. These patients underwent the ICSI procedure with embryo transfer between January 2013 and December 2015, at the Feliccità Fertility Institute, located in the city of Curitiba - Brazil. There was approval of the Research Ethics Committee (CEP) of PUC-PR University, with approval protocol number 1.247.241. The data from the medical records were organized in a spreadsheet. Twenty-six patients who had the procedure canceled due to poor response were removed from the analisys, 46 patients who did not gestate and did not carry out all the exams, and 12 patients who did get pregnant but did not present their results. Thus, our final sample was 238 patients.

### Procedure

The patients were submitted to a controlled hyperstimulation protocol using GnRH agonist (leuprolide acetate - Lupron^®^); or antagonists (cetrorelix acetate - Cetrotide^®^), as well as recombinant ovulation inducers (rFSH - Gonal F^®^).

The cycles were initiated following echographic confirmation of ovarian conditions suitable for ovarian stimulation (absence of cystic masses in ovaries, as well as absence of endometrial pathologies). In the agonist protocol, this was started on day 21 of the menstrual cycle prior to treatment, or on day 1 of their treatment cycle. Doses were decreased on day 3 of the cycle, when stimulation with recombinant FSH was started. In the protocol with antagonist, this was initiated from the observation of follicle with 13mm upon ultrasound.

The follow-up in both cases was performed by serial transvaginal pelvic ultrasound every two days, adjusting the gonadotrophin dose until the observation of 2 or more follicles above 18mm. At that time, choriogonadotropin alpha (Ovidrel^®^) was given. After 36 hours of this last medication, follicular aspiration was performed to obtain the oocytes. The procedure was performed under sedation, transvaginally, and guided by ultrasound. This day was defined as day 0. On the same date, the collection and preparation of the semen was performed, and then proceeded to ICSI. The number of embryos transferred complied with the Resolution of the Federal Board of Medicine (CFM) nº 2116/2015, and was carried out two or three days after the follicular aspiration, being acompanied by pelvic abdominal ultrasonography.

Luteal phase supplementation was started after the uptake, using natural progesterone at a dose of 50mg, intramuscularly once daily and kept until confirmation of gestation. Pregnancy was confirmed with serum bHCG, collected 16 days after collection.

#### Hormonal dosages

The serum levels of hormones were measured with radioimmunoassays for progesterone and prolactin. Progesterone and prolactin levels are expressed in nanograms per milliliter. The samples were collected in the morning, performed nine days after the collection. Most of the samples are sent to the same clinical laboratory, for the consistency on our samples.

#### Statistical analysis

For statistical analysis, the patients were divided into 2 groups: pregnant and non-pregnant. Subsequent comparisons were made between the groups and their respective hormonal dosages. The data was statistically analyzed using the SPSSV.20.0 software, and the results were expressed as percentage, mean and standard deviation; the variables were compared through the chi-square test, with a significance level of 5% (*p*<0.05).

To examine the predictive value that prolactin and progesterone dosages could have in differentiating between pregnant and non-pregnant women, we used the Receiver Operational Characteristic Curves (ROC). The ROC curve traces the relationship between sensitivity and the rate of false positives at varying hormonal concentrations. The suggested cut-off levels for viability prediction were derived from the ROC curve. Sensitivity and specificity were calculated for each cut-off value.

## RESULTS

In a sample of 238 patients, the pregnancy rate was 40.8% (97 pregnancies), as shown in Table 1.

**Table 1:** Pregnancy rate

	Number	%
Pregnant Women	97	40.8
Not Pregnant Women	141	59.2
Total	238	100

The mean prolactin value found on the day of the hormonal analysis was 25.36 for the pregnant women and 24.85 for the non-pregnant women, and this difference was not considered statistically significant (*p*=0.79). Table 2 shows the results.

**Table 2:** Prolactin values compared between groups

PROLACTIN	Mean (ng/ml)	Standart Deviation	*p*
Pregnant Women	25.36	14.49	0.79
Not Pregnant Women	24.85	14.51	

The mean progesterone level in the pregnant group was 35.12 for pregnant women and 28.24 for non-pregnant women, and this difference was considered statistically significant (*p*=0.02). Table 3 shows the results.

**Table 3:** Progesterone values compared between groups

PROGESTERONE	Mean (ng/ml)	Standart Deviation	*p*
Pregnant Women	35.12	26.15	0.02
Not Pregnant Women	28.24	19.26	

**Figure 1:**
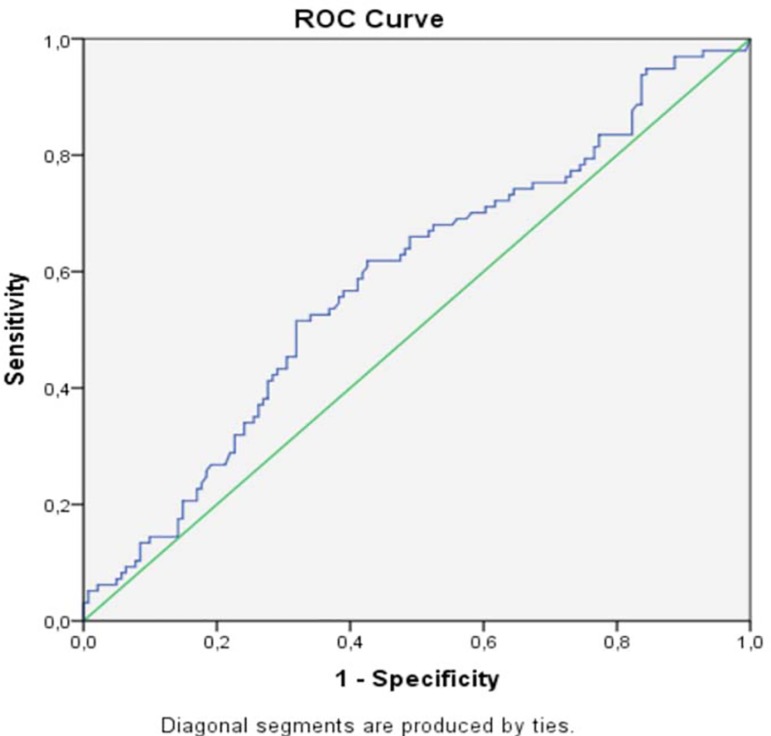
ROC curve for analysis of progesterone levels

Considering that only progesterone presented statistically significant values between the groups, we proceeded with analysis through the ROC curve to establish the progesterone capacity in differentiating between cycles that resulted in gestation or not (Figure 1). With the aid of the ROC curve, the relationship between sensitivity of the method and the presence or not of false positive results in different progesterone concentrations was drawn. Our analysis demonstrated that the ability of progesterone to differentiate between normal and nonviable pregnancies is median, since the area under the curve was 0.585, with a confidence interval between 0.511 and 0.659. The cut-off point indicated by the ROC curve adjustment is 25.95. The sensitivity of this point was 61.9% and the specificity was 57.4%.

## DISCUSSION

Progesterone has been extensively studied in different stages of IVF cycles, with several authors looking for a suitable cut-off point to correct hormonal issues during treatments. Thus, evaluations have been performed at the beginning of stimulation and on the day of hCG administration, to evaluate possible early luteinization of the endometrium. It is known that an embryo implanted in an endometrium with early luteinization has a decrease in its fixation rate. Thus, once this diagnosis is confirmed, it is possible to freeze this embryo or even the oocytes and organize a new cycle for transfer with better conditions for obtaining the pregnancy (Panaino *et al.*, 2017).

Another moment of this hormonal investigation, which motivated this study, is during the luteal phase. Having to wait for the day of pregnancy confirmation brings a high degree of stress to couples. The determination of a progesterone curve that enable us, one week before the β-HCG, to learn about treatment success or failure, would be of great value for both, doctors and patients, during this time.

Progesterone is advocated as one of the best predictors of pregnancy outcome in spontaneous pregnancies (Elson *et al*., 2003). However, in IVF cycles, due to supplementation during the luteal phase, higher levels of progesterone would be expected, even during an unviable pregnancy.

Despite the presence of several luteal bodies in *in vitro* fertilization cycles by ovarian stimulation, the need for luteal phase supplementation is supported by the fact that long pituitary suppression protocols with GnRH analogs are used. As the effect of these products continues up to two or three weeks after oocyte retrieval, the LH pulses would also remain suppressed in the luteal phase, damaging the endogenous production of progesterone and estradiol (Belaisch-Allart *et al*., 1990). Without the LH signal, the corpus luteum may become dysfunctional, which would affect embryonic implantation and decrease pregnancy rates (Beckers *et al*., 2003).

Some authors have evaluated these serum levels. A study by Ioannidis *et al*. (2005) analyzed serum progesterone levels 14 days after follicular aspiration in 442 women who completed IVF cycles or intracytoplasmic sperm injection (ICSI). Among this group, 26% had normal pregnancy at the 8^th^ week of gestation; 18.1% presented unviable pregnancy (biochemistry, ectopic or abortions); and 55.9% did not became pregnant. Although they were receiving rectal progesterone supplements to cover a probable iatrogenic luteal phase deficiency, women with normal and viable pregnancies had higher levels of progesterone, with a median of 135ng/ml, when compared to patients with non-viable gestation who had progesterone at 22.95 ng/ml (*p*<0.001). In this study, patients who did not get pregnant presented progesterone levels of 10.37 ng/ml (*p*<0.001). The study concluded that a progesterone dosage of 32ng/ml yields a diagnostic probability of women who will have a viable pregnancy with a sensitivity of 88.2% and specificity of 84% (Ioannidis *et al.*, 2005).

Another study, carried out by Vicdan & Zeki Isik (2001), evaluated progesterone measurements performed 11 days after oocyte retrieval. In this study, 121 cycles of ICSI treatment were analyzed. No significant differences were found between women who had undergone clinical pregnancy and those who were not pregnant.

Al-Ramahi *et al.* (1999) suggested that a level lower than 14.2 ng/ml in early pregnancy suggests a non-viable gestation. There are reports that progesterone in spontaneous pregnancies is reduced days or weeks before an unfavorable prognosis. Thus, serum progesterone dosage was described as an early detection instrument for abnormal pregnancy (Yeko *et al*., 1987, Hahlin *et al*., 1990). In general, values greater than 20.75 ng/ml were reported for pregnancies with a good prognosis, and levels below 12.57 ng/ml was linked to pregnancy with abnormalities, including ectopic pregnancy and miscarriage (Ioannidis *et al*., 2005).

 Hormonal activity related to the *corpus luteum*, such as serum levels of progesterone and prolactin, could enable the early identification of viable or non-viable pregnancy in spontaneous gestations (Carmona *et al*., 2003). However, these absolute values described above cannot be applied to IVF, because the influence of the GnRH agonist or antagonist on the production of progesterone in the corpus luteum as well as the interference of multiple corpora lutea in serum progesterone values. It is plausible to suppose that, due to the existence of several corpora lutea, high levels of progesterone exist even in unfeasible pregnancies (Yeko *et al.*, 1987; Buck *et al*., 1988; Stovall *et al*., 1989; Peterson *et al*., 1992; Mol *et al*., 1998; Elson *et al*., 2003).

Prolactin is another hormone that can also interfere with the luteal phase. A transient increase in prolactin can be observed in the late follicular phase, both in natural and stimulated cycles, due to the increase in estradiol (Yuen *et al.,* 1979). Since estrogen levels during IVF are much higher than those in a natural cycle, this transient hyperprolactinemia would affect IVF negatively and alter its success rate (Reinthaller *et al*., 1988). This hyperprolactinemia could inhibit the aromatase activity induced by FSH, and it could also lead to luteal phase dysfunction (Dorrington & Gore-Langton, 1981).

Ozaki *et al.* (2001) analyzed serum prolactin on days 7 or 8 after oocyte retrieval in 29 women who would undergo IVF. The patients were broken down into 3 groups: successful pregnancy, early pregnancy loss or non-conception. Prolactin was significantly lower in patients who lost their pregnancies. It was hypothesized that this would be associated whit a delay or defect in endometrial decidualization. This hypothesis is based on the fact that prolactin may come from the maternal decidual endometrium. Although it was unclear whether endometrial prolactin would affect serum levels, it was suggested that higher concentrations of this hormone in the middle of the luteal phase would influence prolactin production by the decidua. But this study has to be analyzed with precaution, because of the small number of patients.

Our study evaluated the potential of prolactin and progesterone measurements in women undergoing IVF/ICSI in patients with progesterone supplementation in the luteal phase. Our results suggest that prolactin measurement cannot be used as a predictive factor of treatment success or failure. However, progesterone assessment on the 9th day after oocyte retrieval proved able to differentiate success or failure in the procedure.

## CONCLUSION

Progesterone assessment may be one of the predictive indicators of wheter *in vitro* fertilization treatments are successful. Values above 26 ng/ml are indicative of good prognosis. Further studies are needed to corroborate the data founded in the present study.
